# Trilateral Attention Network for Real-Time Cardiac Region Segmentation

**DOI:** 10.1109/access.2021.3107303

**Published:** 2021-08-24

**Authors:** GHADA ZAMZMI, SIVARAMAKRISHNAN RAJARAMAN, VANDANA SACHDEV, SAMEER ANTANI

**Affiliations:** 1 National Library of Medicine, National Institute of Health, Bethesda, MD 20892, USA; 2 National Heart, Lung, and Blood Institute, National Institutes of Health (NIH), Bethesda, MD 20892, USA

**Keywords:** Semantic segmentation, localization, attention, fusion, echocardiography

## Abstract

The accurate segmentation of cardiac images into anatomically meaningful regions is critical for the extraction of quantitative cardiac indices. The common pipeline for segmentation comprises regions of interest (ROIs) localization and segmentation stages that are independent of each other and typically performed using separate models. In this paper, we propose an end-to-end network, called Trilateral Attention Network (TaNet), for real-time region localization and segmentation. TaNet has a module for ROIs localization and three segmentation pathways: spatial pathway, handcrafted pathway, and context pathway. The localization module focuses segmentation attention on the desired region while learning the context relationship between different regions in the image. The localized regions are then sent to the three pathways for segmentation. The spatial pathway, which has regular convolutional kernels, is used to extract deep features at different levels of abstraction. The handcrafted pathway, which has hand-designed convolutional kernels, is used to extract a unique set of features complementary to the deep features. Finally, the context (or global) pathway is used to enlarge the receptive field. By jointly training TaNet for localization and segmentation, TaNet achieved superior performance, in terms of accuracy and speed, when evaluated on two echocardiography datasets for cardiac region segmentation.

## I. INTRODUCTION

Medical image segmentation divides the images into different semantic or anatomical regions, from which important quantitative indices can be extracted and used for disease diagnosis. It has been widely applied to various medical imaging modalities, such as X-ray [1], ultrasound [2], and magnetic resonance imaging [3], to delineate anatomical regions for quantitative analysis.

We broadly divide current segmentation approaches into global-based and region-based. Global-based approaches are applied to the entire image while the region-based approaches are applied to a specific region of interest (ROI). The majority of region-based approaches use separate models for ROI localization and semantic segmentation stages. However, combining localization and segmentation stages into a single network is efficient as it prevents unnecessary repetitions of training separate models in isolation [4]–[7]. Further, jointly training the network for localization and segmentation improves the performance as it optimizes segmentation based on localised ROIs.

The spatial and global (context) information are both essential to the semantic segmentation task [8]. Earlier methods for spatial information extraction relied on hand-designed features generated by classical descriptors; e.g., local binary pattern (LBP) [9], [10] and atlases [11], [12]. These hand-designed methods can capture unique statistical, geometrical, or textural features from the images and are optimized for performance and power efficiency [13]. In recent years, deep learning-based methods for segmentation (e.g., fully convolutional networks [14], U-Net [15]) have shown superior performance as they can capture rich low-level details at different levels of abstraction.

Deep learning methods for segmentation can be divided, based on their architectures, into: dilation architecture and encoder-decoder architecture. The dilation architecture uses dilated convolutions to preserve high-resolution feature representations. Examples of current state-of-the-art (SOTA) architectures with dilated convolutions include Deeplabv3 [16] and PSPNet [17]. The encoder-decoder architecture has downsampling and upsampling components and uses skip connections to capture wider context and recover the high-resolution feature representations. Examples of well-known encoder-decoder architectures include U-Net [15] and SegNet [18]. Although these deep learning architectures achieved excellent segmentation performance, the dilation convolutions and the skip connections increase the computational complexity and memory overhead, leading to slow inference speeds. To solve this issue, Yu *et al.* [19], [20] proposed a two-pathway architecture, known as the Bilateral Segmentation Network (BiSeNet), to speed up the segmentation inference time. The first pathway has wide channels and shallow layers to capture the spatial information from the image while the second pathway provides a large receptive field to capture wider context. These two pathways concurrently generate feature representations, which significantly increase the segmentation efficiency. As demonstrated in [19], [20], this conceptual design is significantly faster than dilation and encoder-decoder architectures.

In this work, we extend BiSeNet and propose an efficient end-to-end Trilateral Attention Network (TaNet) for real-time localization and segmentation. Our specific contributions are summarized as follows. First, we use a Spatial Transformer Network (STN) as a module to direct the attention of segmentation to a specific ROI while learning the context relationship between different ROIs. Second, we integrate into BiSeNet [20] a handcrafted pathway (*HP*) with handcrafted-encoded convolutional kernels. This pathway is used to extract, at different levels of abstraction, a unique set of features complementary to the deep features. Third, we present a strategy to jointly train the entire network (localization + segmentation) for cardiac segmentation. Our results, on two echocardiography datasets, show that TaNet achieves impressive real-time results and outperforms current methods for echocardiography segmentation [21], [22].

## II. ECHOCARDIOGRAM DATASETS

We used two echocardiogram datasets to evaluate the proposed network: (A) inferior vena cava (IVC) dataset and (B) parasternal long axis (PLAX) dataset. Both datasets were collected from patients who were enrolled at the Clinical Center in the National Institutes of Health (NIH). All videos in the datasets were acquired using iE33, Sonos, Acuson SC2000, or Epiq 5G ultrasound machines. The use of the de-identified videos was excluded from IRB review per 45 CFR 46 and the NIH policy for the use of specimens/data (ID#18-NHLBI-00686).

### A. IVC DATASET

This dataset contains 268 IVC videos collected from 264 patients. The videos have a spatial resolution of 800 × 600. Each video has ground truth masks, separating IVC from the background, provided by an expert; these masks are verified further by a cardiologist. The images of IVC dataset were resized to 512 × 512 using bicubic interpolation.

### B. PLAX DATASET

PLAX dataset contains 68 B-mode PLAX videos collected from 60 patients. The recorded videos have a spatial resolution of 800 × 1024. Each video has ground truth masks that contain labels for the following cardiac regions: left ventricle (LV), septal wall (SW), posterior wall (PW), right ventricle (RV), and left atrium (LA). These masks are provided by an expert, and verified further by a cardiologist. The images of the dataset were resized to 512 × 512 using bicubic interpolation.

## III. TRILATERAL ATTENTION NETWORK (TaNet)

**FIGURE 1.**
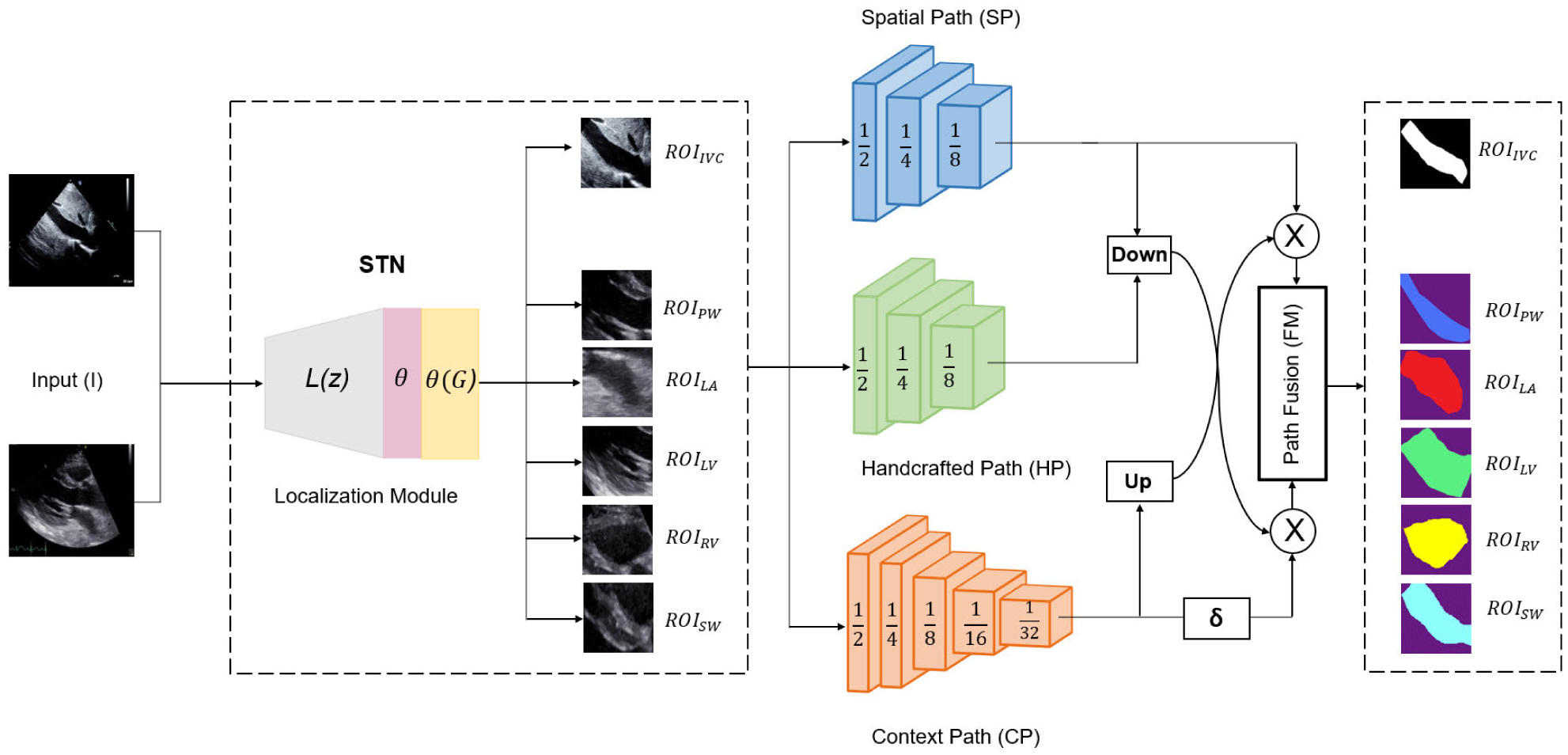
Trilateral Attention Network (TaNet). TaNet, inspired by BiSeNet, has two main components: STN for ROIs localization and segmentation with 3 pathways, spatial (detail) pathway (SP), handcrafted pathway (HP), global or context pathway (CP). The localization module (STN) focuses the segmentation attention to different ROIs. The numbers in the segmentation cubes are the size ratios to the resolution of the input. In the fusion module, we aggregate the feature maps from all pathways. Down, up, ⨂, and *δ* indicate downsampling, upsampling, element-wise product, and sigmoid, respectively.

Figure 1 depicts our proposed network for ROI-based segmentation. TaNet integrates localization into the segmentation by adding the STN module and uses three pathways for segmentation. The entire network is trained end-to-end for localization and segmentation to prevent unnecessary training repetitions and focus on specific ROIs (i.e., cardiac regions) while learning the context relationships among them. In the traditional approach, localization and segmentation models are trained separately as two different stages, which adds unnecessary training repetitions and disregards the relationship between stages.

### A. LOCALIZATION

Although convolutional neural networks (CNNs) are extremely powerful at extracting deep and rich features at different levels, the majority of these networks operate on the whole image and are limited by the spatial invariance of input data. The traditional approach for handling these issues involves using separate models for spatial transformation and localization. In [23], a more efficient approach, called STN, is proposed for applying spatial transformations (e.g., scaling, translation, attention) to the input image or feature map without additional training supervision. STN is a plug-and-play module that can be easily inserted into existing CNNs. It is also differentiable in the sense that it computes the derivative of the transformations within the module, which allows learning the gradients of the loss with respect to the module parameters.

In medical images, it is common that the target ROI occupies a relatively small portion of the image. Hence, considering the entire image for segmentation would add noise caused by irrelevant portions in the background and lead to the segmentation network being biased toward these regions. In this work, we use STN as a method for focusing the attention of the segmentation on a specific ROI while suppressing irrelevant regions. We explain next the main components of STN: the localization network (*L*), grid generator (*G*), and sampler (*S*).

#### 1) LOCALIZATION NETWORK (*L*)

This network takes an image with segmented ROIs (*z*) as input and generates, for each ROI, the spatial transformation parameters (*θ*):

(1) θ=L(z)

where $\theta \in {\mathbb{R}}^{N\times 2\times 3}$, *N* represents the number of cardiac region(s), and *z* is a rough segmentation mask with coarsely labeled cardiac ROIs. In this work, $\theta \in {\mathbb{R}}^{1\times 2\times 3}$ in case of IVC dataset and $\theta \in {\mathbb{R}}^{5\times 2\times 3}$ in case of PLAX dataset.

To generate the rough segmentation mask (*z*) with coarsely labeled ROIs, we follow the approach presented in [24] and use FCN-8 [14] model for coarse segmentation. Providing a coarse segmentation of different ROIs allows the localization network to 1) generate the transformation parameters (*θ*) for these regions and 3) learn the context relationship among different ROIs. The output of the coarse segmentation (FCN-8) is an image (*z*) with coarse semantic labels corresponding to different ROIs.

Given an input image $I\in {\mathbb{R}}^{C_{I}\times H_{I}\times W_{I}}$, where *C*_*I*_ , *H*_*I*_, and *W*_*I*_ represent the image channels, height, and width, respectively, the output of the coarse segmentation model $\left(z\in {\mathbb{R}}^{H_{z}\times {\dot{W}}_{z}}\right)$ can be expressed as:

(2) z=FCN(I)

The predicted rough mask (*z*) is then sent to the localization network (*L*) in Equation 1 to generate the transformation parameter matrix $\left(\theta \in {\mathbb{R}}^{N\times 2\times 3}\right)$. Similar to [24], our localization network (*L*) has eight convolutional layers and a final regression layer to generate *N* × 2 × 3 spatial transformation matrix (*θ*). For each ROI, *θ* is defined as follows:

(3) $$\theta =\left[\begin{array}{ccc} s_{x} & 0 & t_{x}\\ 0 & s_{y} & t_{y} \end{array}\right]$$

where *s*_*x*_, *s*_*y*_, *t*_*x*_, and *t*_*y*_ parameters, which are learned by *L*, allows cropping, translation, and scaling. Note that any cardiac region can be easily added or removed by adjusting *θ*.

#### 2) GRID GENERATOR (*G*)

Given $\theta \in {\mathbb{R}}^{N\times 2\times 3}$, the relevant parts of the image (i.e., *ROI* ∈ {1, 2, …, *N*}) are sampled into a sampling grid *G* of pixels $G_{i}=\left(x_{i}^{t},y_{i}^{t}\right)$ to form *N* output feature maps $V\in {\mathbb{R}}^{C\times H^{\prime }\times W^{\prime }}$, where *C*, *H*′, and *W*′ are the grid’s number of channels, height, and width, which is the same in the input and output. Then, the pointwise affine transformation is computed as:

(4) $$\left(\begin{array}{l} x_{i}^{s}\\ y_{i}^{s} \end{array}\right)=\theta \left(G_{i}\right)=\left[\begin{array}{ccc} s_{x} & 0 & t_{x}\\ 0 & s_{y} & t_{y} \end{array}\right]\left(\begin{array}{c} x_{i}^{t}\\ y_{i}^{t}\\ 1 \end{array}\right)$$

where $\left(x_{i}^{s},y_{i}^{s}\right)$ are the source coordinates, $\left(x_{i}^{t},y_{i}^{t}\right)$ are the target coordinates of the grid in the output feature map, and *θ* is given in Equation 3.

#### 3) BILINEAR SAMPLER (*S*)

To perform the spatial transformation, *θ* for each ROI and the sampling points are sent to a bilinear sampling kernel to produce *N* output maps $V_{1:N}\left(V\in {\mathbb{R}}^{C\times H^{\prime }\times W^{\prime }}\right)$ corresponding to *N* ROIs. Specifically, each coordinate $\left(x_{i}^{s},y_{i}^{s}\right)$ in the sampling grid defines the spatial location in the input where the bilinear sampler is applied to get the value at a particular pixel in the output *V*. This can be written as:

(5) $$V_{i}^{c}=\sum_{n}^{H}\sum_{m}^{W}ROI_{nm}^{c}\text{max}\left(0,1-\left|x_{i}^{s}-m\right|\right)\;\text{max}\left(0,1-\left|y_{i}^{s}-n\right|\right)$$

where $ROI_{nm}^{c}$ is the value at location (*n*, *m*) in channel *c* of the input ROI, and $V_{i}^{c}$ is the output value for pixel *i* at location $\left(x_{i}^{t},y_{i}^{t}\right)$ in channel *c*. Since the bilinear sampling (Equation 5) is differentiable, it allows the gradient loss to flow back to the sampling grid coordinates, and therefore to the transformation parameters *θ* and the localization network (*L*).

This process allows the localization of relevant ROIs in the input image. The localized regions are then sent to the segmentation pathways for pixel-wise prediction as shown in Figure 1. After segmenting the ROIs using, these regions can be remapped to their original positions using a reverse grid transformer (*G*^−1^) [24].

### B. SEGMENTATION

As shown in Figure 1, there are three segmentation pathways: spatial pathway (SP), handcrafted pathway (HP), and context pathway (CP). Each of these pathways extract a unique set of features as described next.

#### 1) SPATIAL PATHWAY (SP)

To extract rich low-level details at a low computational cost, we adopted a shallow spatial pathway that has three convolutional layers with high channel capacity. Specifically, we used three blocks, each containing a 3 × 3 convolutional layer with stride of 2 followed by batch normalization and ReLU activation. The number of filters in the first, second, and third blocks are 64, 64, and 128, respectively. This pathway outputs feature maps that are $\frac{1}{8}$ of the input image size as shown in Figure 1.

#### 2) HANDCRAFTED PATHWAY (HP)

Depending on the medical imaging modality and the application, the standard convolutional kernels can be replaced by handcrafted kernels to extract a unique set of statistical, geometrical, or textural features.

As compared to the handcrafted-based methods, the main strength of deep learning is its ability to learn features at different levels of abstraction, which allows learning complex functions that map the input to the output directly from the data. However, these complex functions may be generic and not directly related to the objects. On the other hand, handcrafted descriptors or kernels are designed to extract unique features (e.g., textural, geometric) that may be different from the ones extracted by deep learning models. For example, the textural features (e.g., LBP) have shown a strong ability to differentiate small differences in texture and topography especially at the boundaries between complex regions with challenging separation. Due to this, handcrafted descriptors are still widely and successfully used in different medical imaging modalities including ultrasound and MRI.

The main limitations of handcrafted features are that they are computed at a single level contrary to the deep features which are extracted at different levels; their performance relies on a set of parameters (usually determined manually); and, therefore they exhibit limited generalizability. In this work, we aim to address these issues by integrating handcrafted kernels into CNN and learning their parameters during training. Specifically, being able to extract, at different levels, a unique set of textural features that are complementary to the deep features while generalizing the parameters of handcrafted kernels in a learnable framework is the main motivation of creating a custom handcrafted-encoded CNN pathway for segmentation. Similar to the SP, we add a HP pathway with three convolutional blocks, but replace the standard convolutional filters with LBP filters. These LBP-encoded kernels are used to extract rich texture features from the echo images.

##### a: FORMULATION

LBP [25] is a theoretically simple and computationally efficient method for summarizing the local texture of an image. This method computes the texture pattern around a central pixel in a local neighborhood by comparing the intensity of the neighborhood pixels (*p*_*i*_) with the central pixel (*p*_*c*_), and assigning a value of 1 if *p*_*i*_ *> p*_*c*_ and 0 otherwise. Then, LBP code is computed by mapping the binary digits to a decimal number using a base of 2. These aggregated LBP codes characterize the image’s texture. Mathematically, the standard LBP is formulated as follows:

(6) $$y_{map}=\sum_{i=1}^{8}\sigma \left(b_{i}*x_{vec}\right)$$

where *x*_*vec*_ is the vectorized input image, *b*_*i*_ are the sparse filters, *σ* is the non-linear binarization function (e.g., Heaviside step), and *y*_*map*_ is the resulting LBP feature map. This formulation (Equation 6) has all the components of the standard convolutional layers. Hence, it can be used to formulate a LBP block with two convolutional layers [26]. The first layer has *m* fixed convolutional filters with non-learnable weights while the second layer has learnable convolutional filters of size 1 × 1.

The first layer is used to generate LBP feature maps as follows. First, the input image (*x*_*vec*_) is processed by *m* predefined convolutional filters (anchor weights) *b*_*i*_, *i* ∈ [*m*] to generate *m* difference maps; i.e., *m* represents the number of LBP filters. Then, these maps are activated using differentiable and non-linear activation functions (e.g., ReLU) to generate *m* bitmaps. Finally, the generated bitmaps are linearly combined by *m* learnable weights *V*_*i*_ ∈ [*m*] to generate the final LBP feature map. This feature map is sent to the next layer. Mathematically, a LBP feature map can be expressed as [26]:

(7) $$y_{map}=\sum_{i=1}^{m}\left(\sum \left(b_{i}*x_{vec}\right)\right)\cdot v_{i}$$

where *y*_*map*_ and *x*_*vec*_ are the output and input images or feature maps, respectively; *m* is the number of predefined convolutional filters, *b*_*i*_ is the 2-sparse convolutional filters, *σ* is the non-linear activation function, and *V*_*i*_ is the learnable weights *V*_*i*_ ∈ [*m*]. To compute the weighted sum of the activations in Equation 7, a convolution operation with filters of size 1 × 1 is used in the second layer. To summarize, each LBP block has two layers, where the first layer has *m* unlearnable convolutional filters followed by learnable filters (1 × 1) in the second layer.

##### b: LEARNABLE PARAMETERS

Each LBP block has a significantly lower number of learnable parameters as compared to the standard convolutional layer[26]. Specifically, the number of learnable parameters in the LBP layer (with the 1 × 1 convolutions) are significantly less than those of a standard convolutional layer for the same size of the convolutional kernel and number of input and output channels. As discussed in [26], LBP encoded CNN has a significantly lower number of parameters as compared to the regular convolutional CNN. Numerically, the parameters are reduced by 9*x*, 25*x*, 49×, 81×, 121×, and 169× for 3 × 3, 5 × 5, 7 × 7, 9 × 9, 11 × 11, and 13 × 13 convolutional filters, respectively.

##### c: LBP BLOCKS

In this work, we propose to integrate a lightweight handcrafted (LBP) pathway to encode unique texture information. As shown in Figure 1, this pathway has three LBP blocks. Each LBP block has a layer with fixed anchor weights (*m*) followed by a second layer with learnable convolutional filters of size 1 × 1. We generated the anchor weights stochastically with different ranges of sparsity. Similar to propagating gradients through layers with learnable and unlearnable parameters (e.g., ReLU, Max Pooling, etc.), the entire path can be trained by back propagating the gradients through the anchor weights as well as the learnable weights. In other words, we leave the anchor weights unaffected and only update the weights of the learnable filters. Empirical results demonstrated that CNNs with LBC layers (non-learnable and learnable) performs comparably to regular CNNs while enjoying significant savings in terms of memory and parameters.

Before proceeding, it is important to note that although we only explored LBP-encoded pathway due to the superiority of LBP in texture analysis especially in ultrasound images, other handcrafted descriptors or kernels (e.g., Gabor) can be easily integrated to the handcrafted pathway following the above formulation.

#### 3) CONTEXT PATHWAY (CP)

Since the size of the receptive field highly impacts the performance of segmentation, several methods (e.g., spatial pyramid pooling [27]) have been proposed to obtain sufficient receptive fields. These methods have high computational complexity and memory consumption.

Inspired by [19], we used a lightweight model (i.e., *Xception*) for fast-downsampling of the feature map to obtain a sufficient receptive field and encode high level context information. Then, we attached a global average pooling on the tail of the lightweight model to provide the maximum receptive field with global context information. Finally, the output of the global average pooling is up-sampled as shown in Figure 1. This pathway rapidly captures high-level context information.

To summarize, the segmentation component of TaNet has three pathways for extracting unique sets of low-level (SP), textural (HP), and context (CP) information. As SP and HP pathways have only three layers, they are not computationally intensive. The CP pathway uses a lightweight model for rapid down-sampling. All the three pathways extract features concurrently, which further increases the network efficiency.

#### 4) PATHWAYS FUSION

Each of the aforementioned pathways extracts different feature representations. The spatial and handcrafted pathways extract low-level details and textural features at different levels, while the context pathway extracts high-level semantic information. Therefore, the simple summation of these feature representations can degrade the performance and complicate the network’s optimization.

To efficiently combine the outputs of the pathways, we first concatenate the output features of each pathway and then use batch normalization to balance the different scales of the features. The concatenated features are combined into a single feature vector (*v*_*concat.*_). This feature vector is sent to a global pooling followed by a convolutional layer (1 × 1), ReLU activation, convolutional layer (1 × 1), and finally Sigmoid function to generate the weight vector *w*_*concat.*_. This weight vector is used to re-weight the concatenated feature vector (*v*_*concat.*_) as follows:

(8) $$v_{\text{output}}=v_{\text{concat}.}*w_{\text{concat}.}+v_{\text{concat}.}$$

### C. LOSS FUNCTION

The loss function of the entire TaNet network can be defined as follows:

(9) $$L=\frac{1}{N}\sum_{i}^{N}L_{Seg}\left(ROI_{i},ROI_{i}^{gt}\right)$$

where *ROI*_*i*_, *i* ∈ {1, 2, …, *N*} represents the pixel-wise prediction for each ROI, $ROI_{i}^{gt}$ represents the corresponding ground truth, and *N* represents the total number of ROIs. Similar to [19], our segmentation loss (*L*_*Seg*_) consists of principal and auxiliary loss functions. The principal loss function is used to supervise the output of the whole network while the auxiliary functions are used to supervise the output of the context pathway. Mathematically, the segmentation loss function (*L*_*Seg*_) is defined as [19]:

(10) $$L_{seg}(Y,W)=L_{p}(Y,W)+\alpha_{1}L_{aux{\;}_{1}}\left(Y_{1},W\right)+\alpha_{2}L_{aux{\;}_{2}}\left(Y_{2},W\right)$$

where *L*_*p*_ is the principal loss (softmax) function, *Y* is the final segmentation for each ROI, *W* is the learnable parameters, $L_{aux_{1}}$ and $L_{aux_{1}}$ are the auxiliary loss functions (softmax) for the context pathway, *Y*_1_ and *Y*_2_ represent the output features from CP. To balance the weight of the loss functions, we empirically set *α*_1_ and *α*_2_ to 1.

### D. NETWORK TRAINING

We trained TaNet in two stages: pre-training and fine-tuning.

#### 1) PRE-TRAINING

**FIGURE 2.**
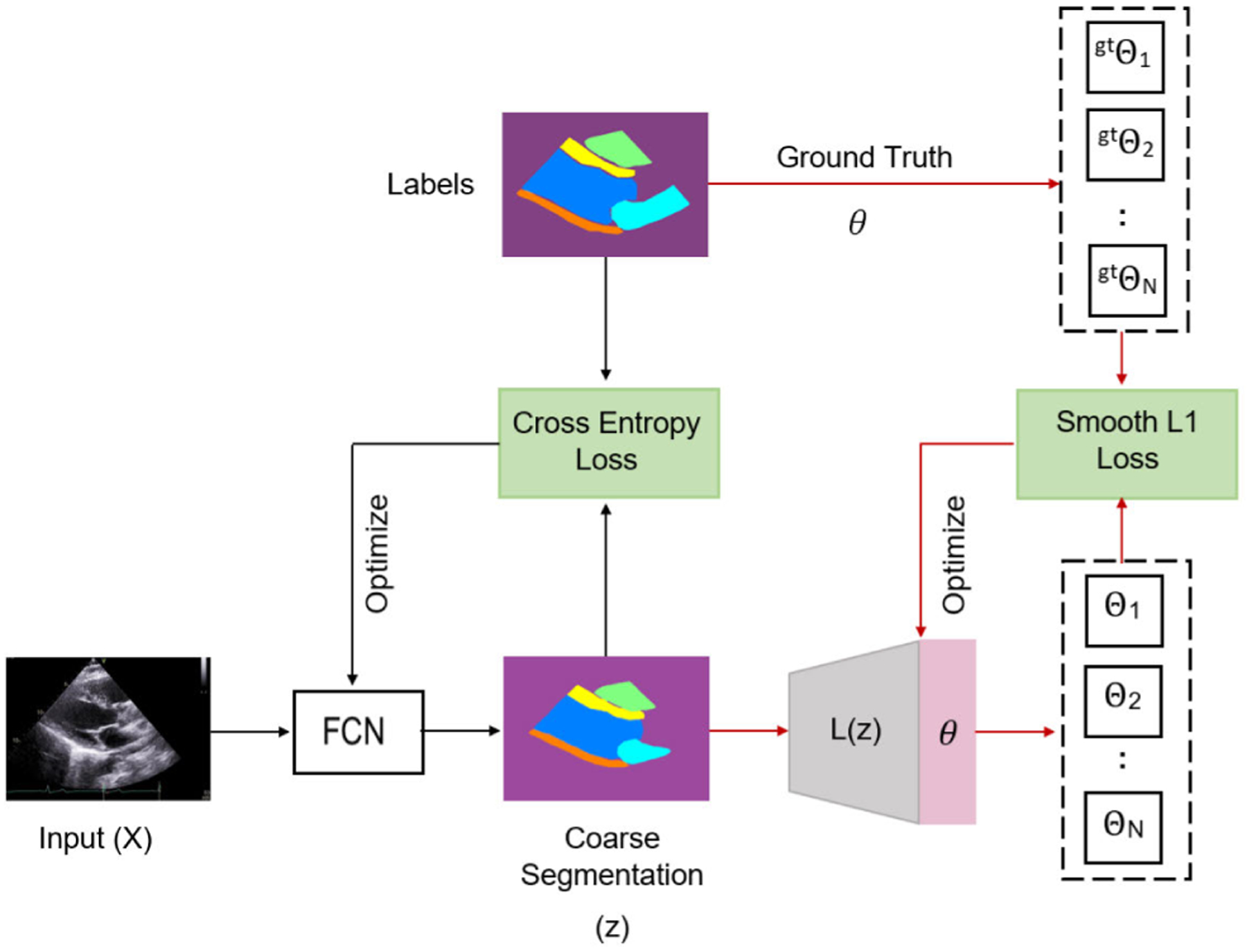
Pre-training steps. The black arrows indicate the first step which involves training a coarse segmentation model while the red arrows indicate the second pre-training step of learning ROIs locations.

As shown in Figure 2, this stage has two steps: 1) pre-training the coarse segmentation model (*FCN*) and 2) pre-training the localization network (*L*). We trained a coarse segmentation model (*FCN*) to get rough predictions of different ROIs. Then, we trained the localization network (*L*) to generate estimations of *θ*_1:*N*_ . It is important to note that this pre-training stage is performed once to estimate the approximate location of different ROIs.

To generate rough ROIs, the coarse segmentation model (*FCN*) is trained with 16 batch size, a learning rate of 1×10^−3^ that is reduced when the validation loss plateau. We used Adam optimizer to minimize the cross entropy loss between GT masks and the predicted coarse segmentation masks. Then, we used the output of the coarse segmentation (*z*) as input to the localization network (*L*). The localization (*L*) is trained with 16 batch size and 1 × 10^−3^ learning rate to optimize the Smooth L1 loss. The smooth L1 loss, which is commonly used for box regression, is less sensitive to outliers [28]. Our localization network (*L*) aims to minimize the smooth L1 loss between predicted *θ* and ground truth *θ*^*gt*^:

(11) $$L1_{\text{smooth}}=\{\begin{array}{l} \;if\;\left|\theta_{r}-\theta_{r}^{gt}\right|<1\Rightarrow 0.5{\left(\theta_{r}-\theta_{r}^{gt}\right)}^{2}\\ \;\text{otherwise}\;\Rightarrow \left|\theta_{r}-\theta_{r}^{gt}\right|-0.5 \end{array}$$

where *θ*_*r*_ and $\theta_{r}^{gt}$ are the predicted and ground truth transformation matrices for a specific region *r* ∈ {1, 2, …, *N*}. The ground truth transformation matrix is calculated for each region $\left(\theta_{r}^{gt},r\in \{1,2,\ldots ,N\}\right)$ as described in [24].

#### 2) END-TO-END FINE-TUNING

With the pre-trained parameters (stage 1) loaded, we fine-tuned the entire network end-to-end. The entire network is trained for 100 epochs with 16 batch size. We used 1 × 10^−3^ learning rate and Adam optimizer to update the network parameters. The optimization goal is to minimize the loss (Equation 9) between ROIs prediction (*ROI*_*i*_) and ROIs *gt* ground truth labels $\left(ROI_{i}^{gt}\right)$.

## IV. EXPERIMENTS

We conducted all experiments using Pytorch and performed training and inference on the NVIDIA GTX1080Ti GPU. In all experiments, we used Talos [29] for selecting the hyperparameters.

We trained two TaNet networks, one for IVC dataset TaNet_*IVC*_) and another for PLAX dataset (TaNet_*PLAX*_). We used 80% of IVC dataset and 70% of PLAX dataset as the training set (subject-wise). These sets are further divided into a training set for coarse segmentation (20%) and a training set for fine segmentation (60%). To enlarge the training sets, we applied the following operations: random rotation (−15° to +15°), horizontal and vertical shift (−0.25,0.25), scale [0.75, 1, 1.25], and horizontal and vertical flip. Finally, the trained fine segmentation networks are evaluated using the remaining 20% (subject-wise) of IVC and PLAX datasets.

For each model (i.e., TaNet_*IVC*_ and TaNet_*PLAX*_), we performed two ablation experiments to validate the impact of TaNet components, namely the attention module (STN) and handcrafted pathway (HP). We also compared the performance of TaNet to state-of-the-art models for segmentation including the baseline BiSeNet [19], FCN [14], and UNET [15]. We compared the automated segmentation against the human segmentation and reported the performance in terms of prediction (intersection over union [IoU] and Dice or F1 score) and inference speed (frame per seconds [FPS]).

### A. ABLATION FOR STN MODULE

To evaluate the impact of STN, we integrated STN module to the baseline Bisenet [19], FCN [14], and UNET [15] and reported the segmentation results for each cardiac region as shown in Table 1 and Table 2. We applied the segmentation to 512 × 512 images and reported the results using the average IoU and F1 score, which are averaged over the test samples. As can be seen in Table 1 and Table 2, integrating STN into segmentation models slightly decreases the inference speed. It, however, improves cardiac region segmentation in most cases. Note that the improvement in segmentation is higher in case of PLAX dataset (Table 2) as compared to IVC dataset (Table 1). This might be attributed to the simpler structure of IVC images as compared to PLAX images with overlapped cardiac regions. Nonetheless, these results demonstrate STN’s ability to increase the segmentation performance by focusing the attention on the desired region while learning the context relationship of different regions in the image.

### B. ABLATION FOR HANDCRAFTED PATHWAY

To evaluate the impact of integrating LBP-encoded kernels (handcrafted pathway) on the segmentation performance and speed, we replaced the classical kernels in FCN8, UNET, and BiSeNet with LBP-encoded kernels. Table 1 (IVC) and Table 2 (PLAX) show that using LBP-encoded layers achieved better performance as compared to the classical convolutional kernels while increasing the inference speed. Note that using LBP-encoded layers significantly improves the performance of SW and PW regions (Table 1). Due to the similarity between the anterior and posterior walls around LV, the accurate segmentation of LV walls is challenging. However, combining the textural features with the rich low-level features improved the segmentation of these walls. These results suggest the ability of the handcrafted pathway (integrated with textural kernels) to differentiate small differences in texture at the boundaries between complex regions with challenging separation.

**FIGURE 3.**
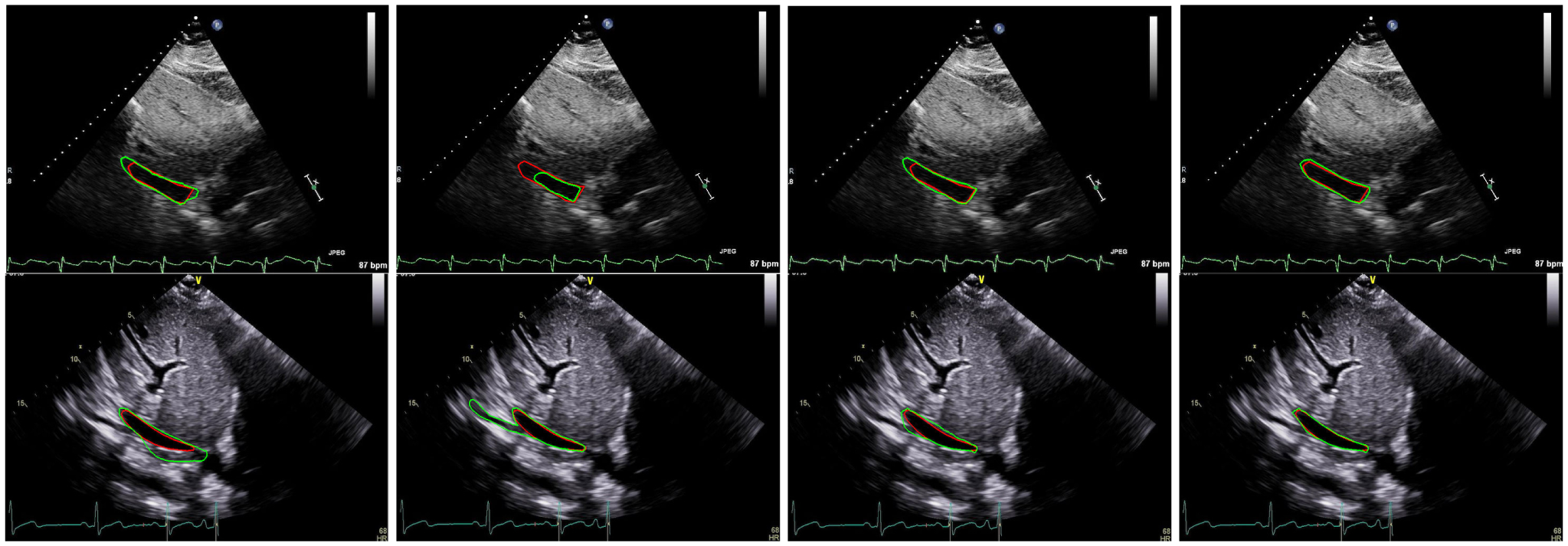
IVC segmentation: ground truth contour (red) and the automated contour (green) generated by FCN8 (1^*st*^ column), UNET (2^*nd*^ column), BiSeNet (3^*rd*^ column), and TaNet (4^*th*^ column).

**FIGURE 4.**
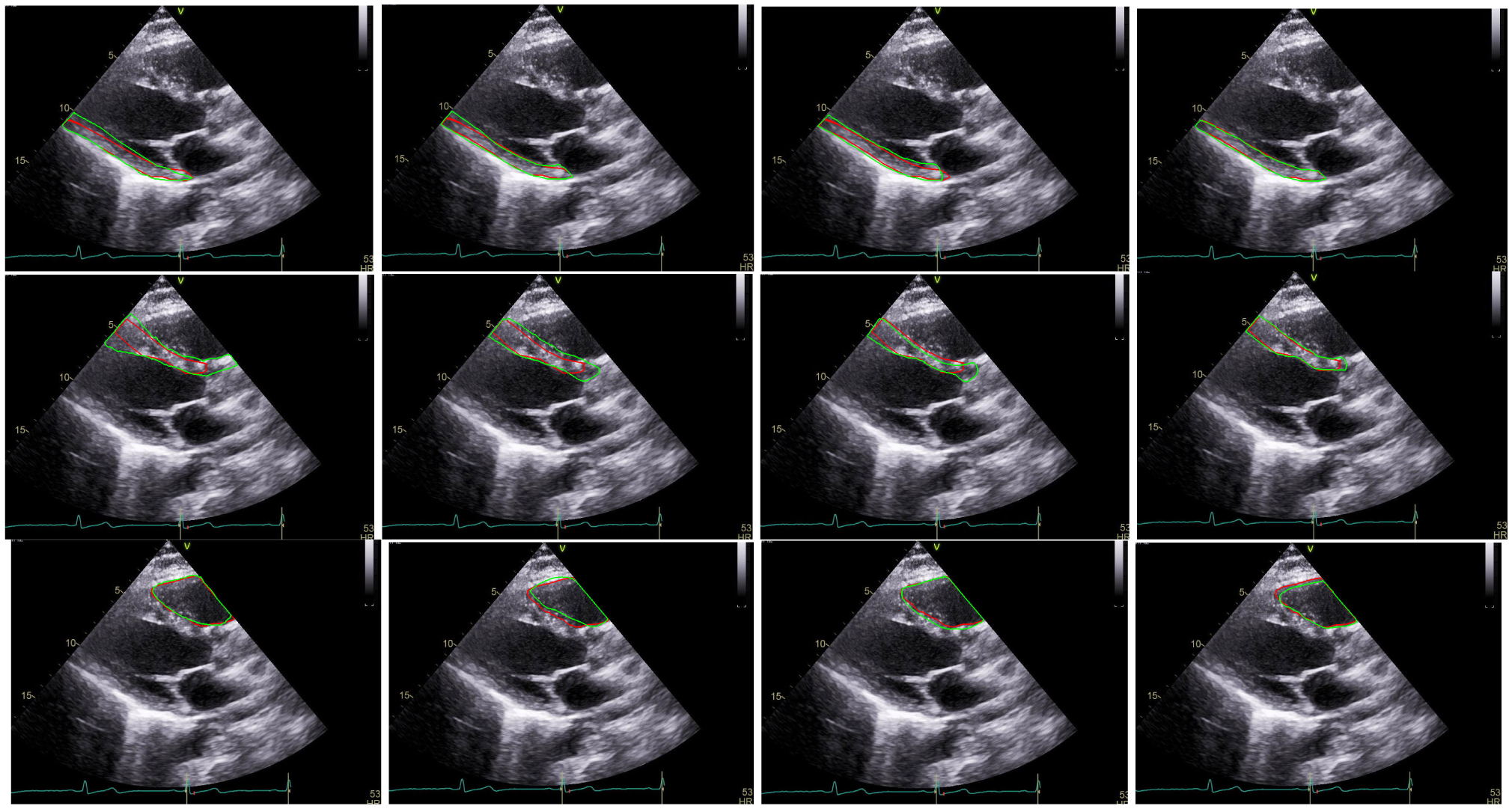
PLAX segmentation: Ground truth contour (red) and the automated contour (green) generated by FCN8 (1^*st*^ column), UNET (2^*nd*^ column), BiSeNet (3^*rd*^ column), and TaNet (4^*th*^ column). 1^*st*^ row: PW, 2^*nd*^ row: LV, 3^*rd*^ row: SW, 4^*th*^ row: RV, and 5^*th*^ row: LA.

**FIGURE 5.**
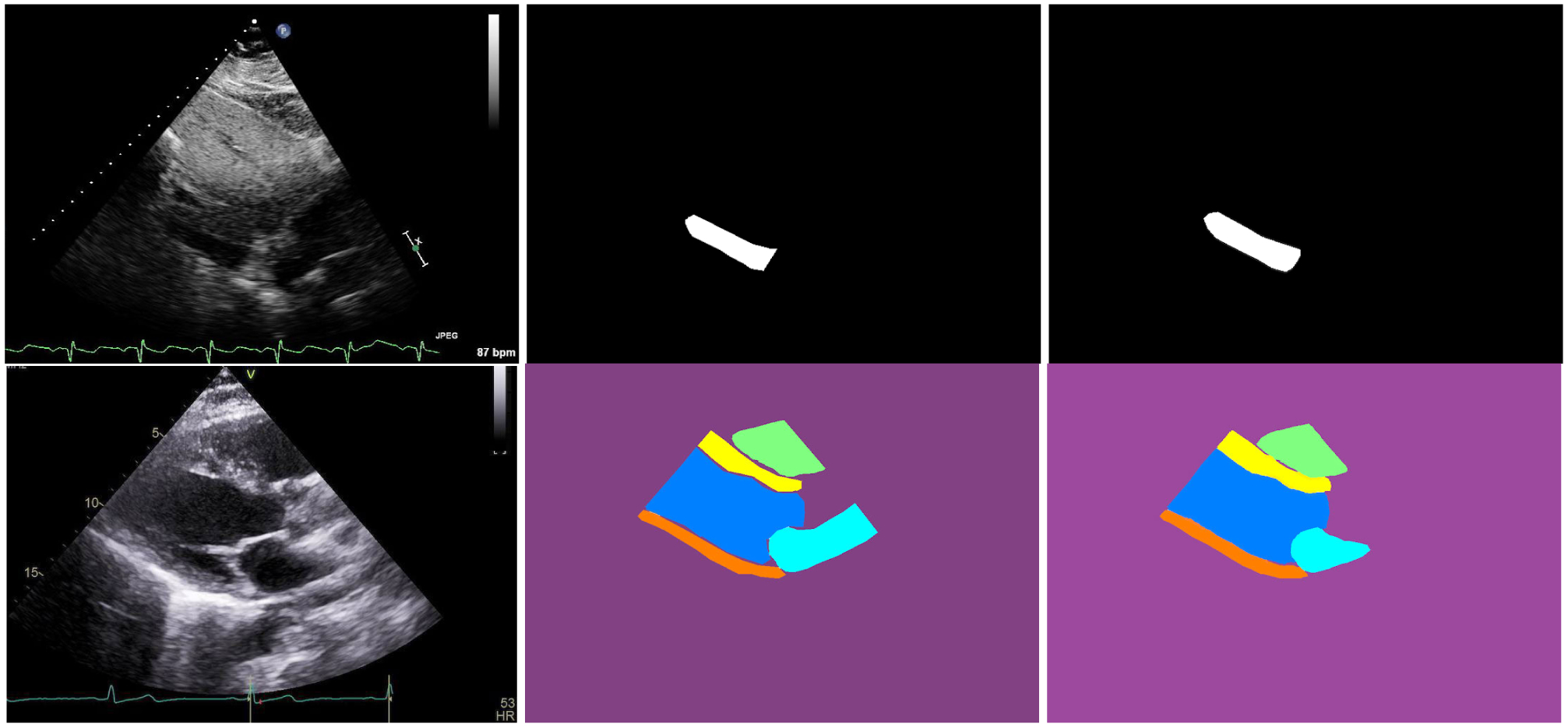
IVC (1^*st*^ row) and PLAX (2^*nd*^ row) segmentation: original image (1^*st*^ column), ground truth mask (2^*nd*^ column), and mask predicted by TaNet (3^*rd*^ column). Green ROI (RV), yellow ROI (SW), dark blue ROI (LV), orange ROI (PW), and light blue ROI (LA).

The last rows of Table 1 and Table 2 present the performance of TaNet (STN, HP, SP, CP) as compared to the baseline BiSeNet (*SP* + *CP*) [19], FCN-8 [14] and UNET [15]. The green cells indicate that the proposed TaNet achieved significantly (*p <* 0.05) higher performance as compared to the baseline models. Note that TaNet has a slightly lower speed as compared to BiSeNet [19]. However, this speed is still efficient for real-time medical image analysis. Figure 3 and Figure 4 show segmentation examples of TaNet and baseline models from IVC (Figure 3) and PLAX (Figure 4) datasets, respectively. Figure 5 shows the ground truth masks and predicted masks for images from IVC and PLAX datasets. As shown in the figures, TaNet achieved excellent performance segmenting different cardiac regions. This is attributed to TaNet’s ability to focus on specific regions while using texture kernels to capture slight differences between complex regions. We believe this conceptual design of TaNet is suitable for medical images segmentation as it allows independent analysis for each region separately as well as all regions together.

### C. COMPARISON WITH STATE-OF-THE-ARTS

The majority of segmentation methods for echocardiography segmentation rely on different extensions of UNET [30], [31] and FCN [32]. Other recent methods for echocardiography segmentation include Res-U [22] and CA-Net [33]. In this section, we compare the proposed network to recent segmentation models [22], [33] for echocardiography segmentation.

Table 3 provides a performance comparison of TaNet and state-of-the-art segmentation models using our IVC and PLAX datasets. From the table, we can observe that TaNet achieved the best average IoU and F1 score for both PLAX and IVC datasets. Further, TaNet achieved the second best inference speed. Note that the lower speed of TaNet (as compared to BiSeNet) might be attributed to the integration of the STN-based localization component and the additional handcrafted pathway for extracting rich textural features. However, the results showed that the integration of these two components enhanced the segmentation performance. From Table 3, we can conclude that the proposed network achieved excellent real-time performance and outperformed existing models for echocardiography segmentation.

## V. CONCLUSION

This work proposes a network (TaNet) for real-time ROI-based segmentation. TaNet achieved superior performance when evaluated using two echocardiography datasets for cardiac region segmentation. This superiority is attributed to 1) the ability of STN to focus the segmentation attention on specific ROIs while learning the context relationship between them and 2) the use of the handcrafted pathway with texture kernels along with the spatial pathway allowing the extraction of rich low-level and textural features. In addition to the superior performance, TaNet has a relatively small size and high inference speed, which allows the deployment of the network on platforms with limited computational resources and enables real-time segmentation in clinical practice and point-of-care testing. While our empirical results are promising, we plan to further evaluate TaNet on other imaging modalities including CT and MRI. To benefit the research community, we make our code available, and invite researchers to contribute to this effort.

## Figures and Tables

**TABLE 1.** Results of ablation experiments to evaluate the impact of STN and LBP-encoded kernels on segmentation. The results are reported on the testing set of IVC dataset. Bold numerical values stand for best performance. Green cells indicate statistical difference between TaNet and baseline models, without STN (✕) and LBP (✕).

Model	STN	LBP	IVCI		FPS
			IoU	F1	
FCN8	✕	✕	0.83 ± 0.01	0.91 ± 0.07	6.25
	✓		0.86 ± 0.17	0.93 ± 0.22	4.1
		✓	0.84 ± 0.25	0.92 ± 0.16	8.2
UNET	✕	✕	0.86 ± 0.12	0.93 ± 0.05	10.4
	✓		0.88 ± 0.08	0.93 ± 0.15	6.8
		✓	0.86 ± 0.09	0.92 ± 0.12	12.7
BiSeNet	✕	✕	0.92 ± 0.09	0.95 ± 0.11	104.8
	✓		0.94 ± 0.05	0.97 ± 0.07	89.7
		✓	0.86 ± 0.11	0.92 ± 0.17	123.5
TaNet	✓	✓	**0.96** ± 0.03	**0.98** ± 0.05	94.3

**Table 2.** Results of ablation experiments to evaluate the impact of STN and LBP-encoded kernels on segmentation. The results are reported on the testing set of PLAX (LV, RV, LA, SW, and PW) dataset. Bold numerical values stand for best performance. Green cells indicate statistical difference between TaNet and baseline models, without STN (✕) and LBP (✕).

Model	STN	LBP	LV		RV		LA		SW		PW		FPS
			IoU	F1	IoU	F1	IoU	F1	IoU	F1	IoU	F1	
FCN8	✕	✕	0.87 ± 0.03	0.93 ± 0.05	0.82 ± 0.09	0.91 ± 0.19	0.83 ± 0.17	0.91 ± 0.13	0.86 ± 0.05	0.93 ± 0.03	0.77 ± 0.012	0.87 ± 0.16	5.5
	✓		0.93 ± 0.09	0.96 ± 0.08	0.80 ± 0.13	0.89 ± 0.06	0.89 ± 0.16	0.94 ± 0.08	0.86 ± 0.10	0.93 ± 0.02	0.76 ± 0.14	0.87 ± 0.03	3.8
		✓	0.88 ± 0.14	0.93 ± 0.20	0.80 ± 0.04	0.89 ± 0.10	0.84 ± 0.22	0.91 ± 0.13	0.89 ± 0.07	0.94 ± 0.09	0.73 ± 0.16	0.84 ± 0.18	9.3
UNET	✕	✕	0.85 ± 0.18	0.91 ± 0.08	0.81 ± 0.13	0.89 ± 0.02	0.88 ± 0.13	0.93 ± 0.11	0.87 ± 0.04	0.93 ± 0.03	0.79 ± 0.02	0.88 ± 0.01	4.8
	✓		0.90 ± 0.03	0.95 ± 0.05	0.86 ± 0.09	0.92 ± 0.10	0.91 ± 0.17	0.95 ± 0.13	0.90 ± 0.05	0.94 ± 0.03	0.81 ± 0.19	0.89 ± 0.16	3.1
		✓	0.87 ± 0.02	0.93 ± 0.07	0.88 ± 0.11	0.94 ± 0.13	0.87 ± 0.20	0.93 ± 0.09	0.83 ± 0.15	0.90 ± 0.004	0.81 ± 0.01	0.89 ± 0.13	7.2
BiSeNet	✕	✕	0.89 ± 0.01	0.94 ± 0.09	0.85 ± 0.03	0.92 ± 0.01	0.89 ±	0.94 ± 0.02	0.85 ± 0.10	0.92 ± 0.16	0.77 ± 0.23	0.87 ± 0.20	100.1
	✓		0.94 ± 0.11	0.97 ± 0.05	0.90 ± 0.12	0.94 ± 0.09	0.92 ± 0.17	0.96 ± 1.10	0.90 ± 0.04	0.95 ± 0.01	0.78 ± 0.15	0.87 ± 0.19	90.4
		✓	0.91 ± 0.09	0.95 ± 0.11	0.86 ± 0.22	0.92 ± 0.17	0.93 ± 0.08	0.96 ± 0.02	0.90 ± 0.15	0.94 ± 0.10	0.89 ± 0.24	0.92 ± 0.21	135.4
TaNet	✓	✓	**0.95** ± 0.004	**0.98** ± 0.01	**0.91** ± 0.05	**0.95** ± 0.02	**0.93** ± 0.04	**0.97** ± 0.06	**0.93** ± 0.07	**0.96** ± 0.01	**0.88** ± 0.13	**0.93** ± 0.17	84.6

**TABLE 3.** Performance comparison of the proposed TaNet and state-of-the-art segmentation models. Bold values represent best performance and inference speed (FPS).

Model	mloU_*PLAX*_	IoU_*IVC*_	F1_*IVC*_	FPS (PLAX-IVC)
TaNet	**0.907**	**0.944**	**0.981**	84.6–94.3
BiSeNet	0.854	0.923	0.959	**100.1–104.8**
CA-Net	0.903	0.915	0.955	8.1–11.5
Res-U	0.846	0.846	0.916	2.5–8.3
UNET	0.854	0.884	0.938	4.8–10.4
FCN8	0.831	0.833	0.908	5.5–6.3
